# Exploring the determination of the standard rate constant in electrochemical metal deposition: theory and experiment

**DOI:** 10.1039/d5sc05636e

**Published:** 2025-10-08

**Authors:** Rania Saad Guermeche, Abed Mohamed Affoune, Sabrina Houam, Imene Atek, Christine Vautrin-Ul, Mouna Nacef, Mohamed Lyamine Chelaghmia, Hubert H. Girault, Craig E. Banks, Ilhem Djaghout, Jacques Bouteillon, Jean Claude Poignet

**Affiliations:** a Laboratory of Industrial Analysis and Material Engineering, Department of Process Engineering, University 8 May 1945 Guelma BP 401 Guelma 24000 Algeria affoune2@gmail.com; b Laboratory of Process Engineering for Sustainable Development and Health Products, Preparatory Classes Department, National Polytechnic School of Constantine Constantine 25000 Algeria; c Laboratoire ICMN Interfaces, Confinement, Matériaux et Nanostructures, UMR7374, Université d‘Orléans–CNRS 1b rue de la Férollerie 45071 Orléans Cedex 2 France; d Laboratoire d'Electrochimie Physique et Analytique, École Polytechnique Fédérale de Lausanne, EPFL Valais Wallis Rue de l'Industrie 17, Case Postale 440 CH-1951Sion Switzerland; e Faculty of Science and Engineering, Manchester Metropolitan University Dalton Building, Chester Street Manchester M1 5GD UK; f Laboratoire d’électrochimie et de physicochimie des matériaux et des interfaces 1130 rue de la Piscine 38402 Saint Martin d'Hères France

## Abstract

A detailed investigation of the electrochemical metal deposition was carried out using both simulation and experimental cyclic voltammetry (CV). Kinetic curves were developed to relate peak-to-peak potential separation (Δ*E*_p_) to the cathodic charge transfer coefficient (*α*) and the standard rate constant (*k*^0^). From these curves, interpolation equations were derived to estimate *k*^0^, taking into account the effect of the transfer coefficients sum (*α* + *β*), whether equals to or different from 1. The validity of the equations was confirmed through the reduction of silver, copper and rhenium ions in various electrolytes, yielding *k*^0^ values of 14.51 × 10^−6^ m s^−1^ for Ag^+^/Ag, 5.98 × 10^−7^ m s^−1^ for Cu^+^/Cu and 10.59 × 10^−8^ m s^−1^ for Re^6+^/Re. According to the Matsuda–Ayabe criteria for assessing electron-transfer reversibility, the Ag^+^/Ag and Cu^+^/Cu redox couples are regarded as quasi-reversible, while the Re^6+^/Re couple is classified as irreversible. The simulated CVs showed strong agreement with experimental results.

## Abstract 2

Interpolation equations from kinetic curves enable *k*^0^ extraction from Δ*E*_p_ in electrodeposition, accounting for *α* + *β* effects and experimental validation.

## Graphical abstract

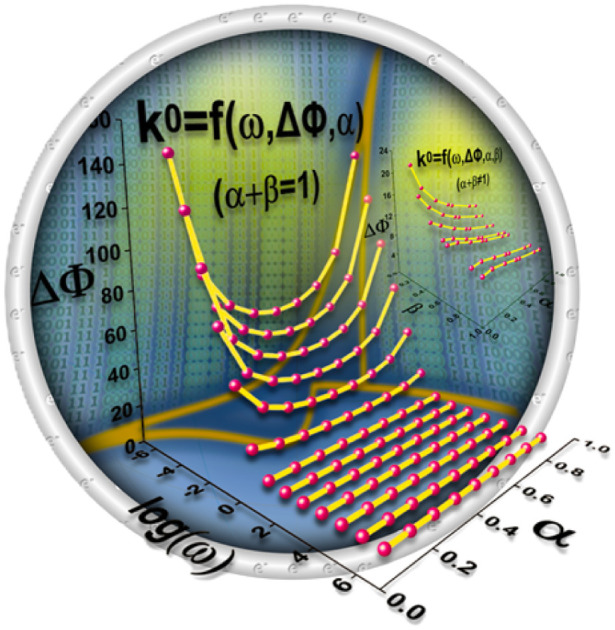

## 1. Introduction

Voltammetric techniques play a crucial role in assessing both the thermodynamic and kinetic aspects of redox processes. Among the different voltammetric methods, cyclic voltammetry is the most frequently used.^1–3^

Linear sweep voltammery (LSV) and cyclic voltammetry (CV) simulations have attracted significant attention from researchers.^3–24^ By developing accurate mathematical models and gaining deep insights into the underlying mechanisms, scientists strive to unravel the complexity of CV responses. The simulation of CV for electrochemical metal deposition was first studied by Berzins and Delahay^10^ for a reversible system, followed by numerous other researchers.^11–13^ Delahay^14^ also conducted studies on irreversible systems. Different works on quasi-reversible systems have been carried out by Atek,^15^ and other investigators.^18,25–28^ Additionally, studies from Saila, Affoune, Avaca and Kanzaki^16,17,29,30^ have explored the electro-oxidation of insoluble species.

The standard heterogeneous electron transfer rate constant (*k*^0^) is a crucial electrochemical parameter, as it provides direct insight into the kinetics of redox reactions.^21,31–34^ Standard rate constant determination is a fundamental scientific concept with significant cross-disciplinary implications, as it provides quantitative insights into reaction mechanisms and speeds across various fields like electrocatalysis, materials science, energy storage and biology.^35–48^ In electrocatalysis, it is used to characterize the activity and efficiency of electrocatalysts for various reactions.^35–37^ in materials science, it aids in understanding the behavior and stability of materials and devices like batteries, electroplatings and sensors.^38–46^ In biology, standard rate constants are crucial for quantifying protein-ligand interactions and enzyme kinetics, helping to understand biological processes like signaling, drug discovery, and the mechanisms of genetic and biochemical reaction.^47,48^

In contrast to soluble–soluble redox couples, for which the standard rate constant (*k*^0^) has been extensively investigated through linear sweep voltammetry (LSV)^6,8^ and cyclic voltammetry studies,^7,49–51^ the case of soluble–insoluble couples has received far less attention, with only a very few studies examining the determination of *k*^0^ using voltammetric techniques. Atek^15^ presented kinetic diagrams as well as their interpolation equations to determine the kinetic rate constant through the development of interpolation equations based on peak current, half-peak width and peak potential kinetic curves, building on the approach initially introduced by Krulic,^26^ where the sum of the cathodic (*α*) and anodic (*β*) charge transfer coefficients equals 1.

As far as we are aware, the standard rate constant for electrochemical metal deposition has not been investigated based on cyclic voltammetry whatever the sum (*α* + *β*) across an extended ranges of Δ*E*_p_ and charge transfer coefficient (*α*). This work investigates how the charge transfer coefficients *α*, *β*, and their sum (*α* + *β*) affect cyclic voltammetry, particularly the peak-to-peak potential separation (Δ*E*_p_) and the accurate determination of the standard rate constant *k*^0^. Kinetic curves and interpolation equations were developed to express Δ*E*_p_ as a function of the dimensionless rate constant (*ω*) and charge transfer parameters. The influence of *α* + *β* on *k*^0^ estimation was analyzed and corrected. Experimental validation was carried out, and findings enable reliable *k*^0^ determination for electrodeposition reactions using cyclic voltammetry peak separation.

## 2. Results and discussion

### 2.1. Metal deposition cyclic voltammograms characteristics

**Fig. 1:**
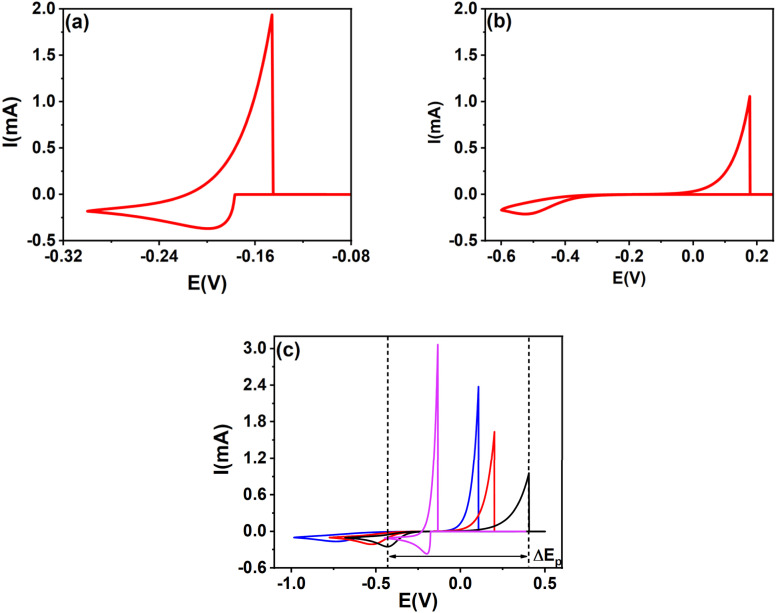
The effect of *ω* = 10^3^ (a), *ω* = 10^−3^ (b), and *α* (c) on soluble-insoluble CVs: *α* = 0.7 (black), *α* = 0.5 (red), *α* = 0.3 (blue) for *ω* = 10^−3^ and *α* = 0.5 (magenta) for *ω* = 10^3^.

Fig. 1 presents theoretical cyclic voltammograms for electrodeposition reaction, illustrating both the effect of the dimensionless rate constant (*ω*) and the cathodic charge transfer coefficient (*α*). The relationship between *ω* and *k*^0^ (
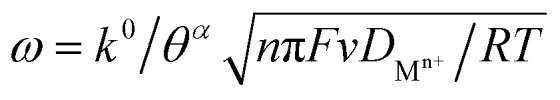
) is provided in the SI (S14). Simulations were performed using the following parameters: *n* = 1, *T* = 298.15 K, *ν* = 0.1 V s^−1^, 
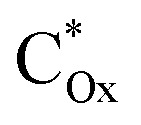
 = 1 mM, *A* = 1 cm^2^, *D* = 1 × 10^−9^ m^2^ s^−1^.

Fig. 1a–c shows that the cathodic peaks of electrochemical metal deposition exhibit an asymmetric convex shape, while the anodic peaks appear sharp and narrow, accompanied by a steep current drop immediately after the peak, and does not exhibit a diffusion-controlled phase. The increase in anodic current is exclusively governed by charge transfer. The rapid current drop indicates the complete oxidation of deposit formed during the forward scan. The separation between the anodic and cathodic peak potentials (Δ*E*_p_) increases with increasing irreversibility; *i.e.* with decreasing rate constant *ω*, and a lower cathodic charge transfer coefficient (*α*).

### 2.2. Electrochemical standard heterogeneous rate constant (*k*^0^) determination: case where *α* + *β* = 1

First, we studied how the switching potential (*E*_λ_) influences the peak-to-peak potential separation (Δ*E*_p_), (see SI). After that, we investigate the combined effect of cathodic charge transfer coefficient and dimensionless rate constant on Δ*E*_p_.

Since the sum of the cathodic and anodic transfer coefficients (*α* + *β*) directly affects peak positions and, by extension, the peak-to-peak potential separation (Δ*E*_p_), its impact is examined in depth in this study. Distinct analyses are conducted for systems where *α* + *β* = 1 and those where this condition is not satisfied, highlighting the differences in electrochemical behavior under each scenario.

The relationship between peak-to-peak potential separations (Δ*E*_p_) and dimensionless peak-to-peak potential separations (Δ*Φ*) is expressed as follows:1
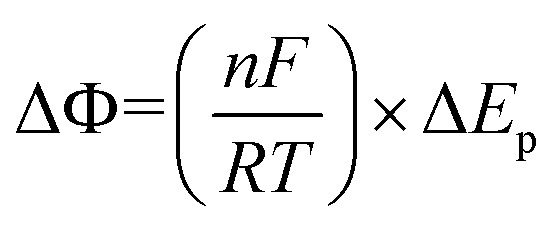


In this study, we investigate a broader range of the dimensionless kinetic rate constant (*ω*), spanning from 10^−6^ to 10^6^, along with cathodic charge transfer coefficient (*α*) values varying between 0.1 and 0.9.

**Fig. 2:**
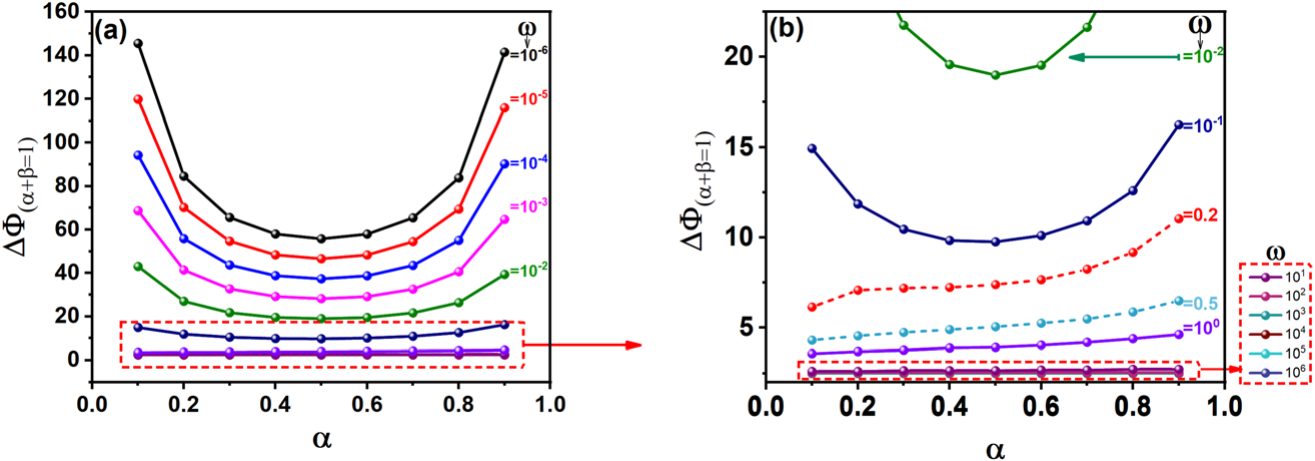
Kinetic curves presenting the effect of the charge transfer coefficient (*α*) and the kinetic rate constant (*ω*) on the peak-to-peak potential separation (Δ*Φ*): (a) 10^−6^ ≤ *ω* ≤ 10^6^, (b) *ω* ≥ 10^−2^.

Fig. 2 shows kinetic curves for electrochemical metal deposition redox systems, where the dimensionless peak-to-peak potential separation (Δ*Φ*) is plotted as a function of the logarithm of the dimensionless rate constant (log *ω*) and the cathodic charge transfer coefficient (*α*). For a known value of *ω*, simulated voltammograms are established for nine values of *α* = [0.1–0.9]. From the file data of each voltammogram, the dimensionless peak-to-peak potential separation (Δ*Φ*) is deduced. After that, the same procedure is repeated for another value of *ω* = (10^−6^, 10^−5^, …, 10^6^).

Throughout this work, Δ*Φ*_(*α*+*β*=1)_ will refer to the peak separation under the condition *α* + *β* = 1. The dimensionless rate constant (*ω*) for metal electrodeposition reactions depends on the initial concentration of the oxidized species as indicated in eqn (S14). The diagrams presented in Fig. 2a and b were calculated with the concentration 
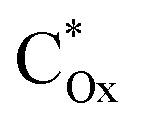
 = 1 mM. However, these diagrams remain valid for any concentration, because the concentration affects only the current and has no influence on the peak potential.

In the range *ω* = 10^−6^ to 10^−1^, Fig. 2a reveals a convex and symmetrical profile, centered around *α* = 0.5. In contrast, Fig. 2b shows that as *ω* increases beyond 10^−1^, the curves become increasingly asymmetric and flatten in shape. When the values of *α* and Δ*E*_p_ are known, the kinetic curves provided above allow for the determination of the dimensionless rate constant *ω*. Once *ω* is obtained, the standard rate constant *k*^0^ can then be calculated using eqn (S14).

As noted earlier, the symmetry of the kinetic curves for *ω* ≤ 10^−1^ supports the use of interpolation. Data fitting was performed using the rational Holliday equation:2
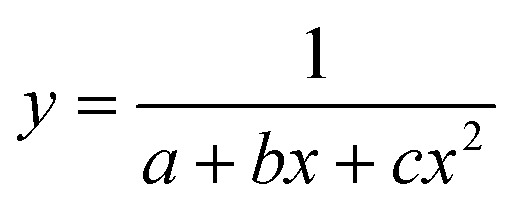
where *y* represents Δ*Φ* and *x* represents *α*. *a*, *b* and *c* are functions of log(*ω*).

The following equation was derived within these parameter ranges:−6 ≤ log(*ω*) ≤ −1, 10 ≤ Δ*Φ* ≤ 1503
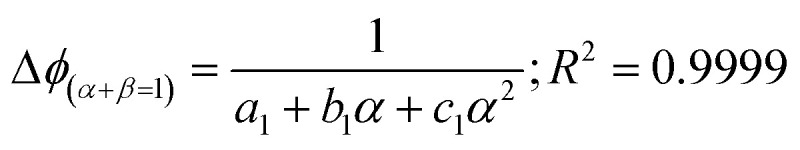
where:

4 *a*_1_ = 0.0012 + 0.352 exp((−log(*ω*))/−0.4862); *R*^2^ = 0.9930

5 *b*_1_ = 0.3045 exp(0.2641 log(*ω*)); *R*^2^ = 0.9930

6 *c*_1_ = −0.3136 exp(0.27414 log(*ω*)); *R*^2^ = 0.9930

When *α* + *β* = 1 and *ω* is in the range *ω* ≤ 10^−1^, *ω* can be accurately extracted using either the interpolation eqn (3) or the corresponding kinetic curves shown in Fig. 2. In contrast, for *ω* values exceeding 10^−1^, the irregular and flattened nature of the curves prevents reliable interpolation. Nonetheless, as depicted in Fig. 2b, the impact of *α* on Δ*E*_p_ becomes negligible in this higher *ω* range. As a result, approximate values of *ω* can still be inferred from the detailed view of the kinetic curves in Fig. 2b.

### 2.3. Electrochemical standard heterogeneous rate constant (*k*^0^) determination: case where *α* + *β* ≠ 1

**Fig. 3:**
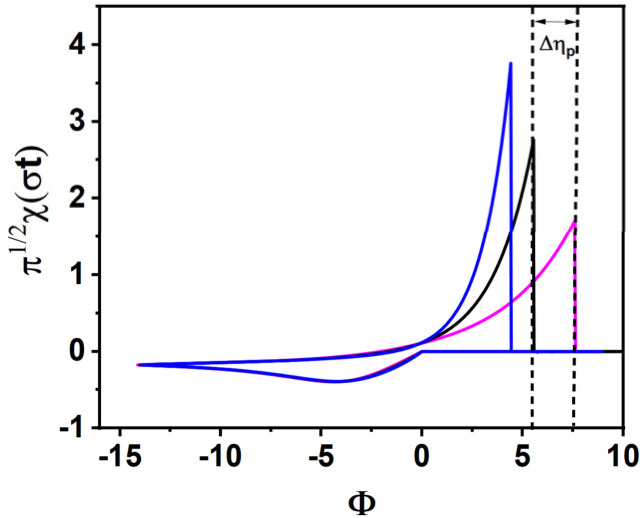
Presentation of dimensionless soluble–insoluble CVs for *ω* = 10^−1^, *α* = 0.5 and *β* = 0.3, 0.5, 0.7.

The electrochemical literature acknowledges that the sum of the charge transfer coefficients (*α* + *β*) can differ from one, either being less than or greater than unity.^52–60^ Chen ^54^explained that in Butler–Volmer theory, the assumption that *α* + *β* = 1 is derived from the concept of microscopic reversibility. However, this condition is only valid at the equilibrium potential. In systems that are quasi-reversible, this assumption does not hold. The Marcus–Hush theory, especially in its asymmetric form, provides an explanation for these discrepancies, relating them to the differing vibrational force constants of the redox species. Additionally, Henstridge^55^ observed that, due to the large peak-to-peak separation, the condition *α* + *β* = 1 can be relaxed without violating the principle of microscopic reversibility. This is because the oxidation and reduction reactions take place at widely differing potentials, placing them in distinct environmental conditions. Suwatchara^52^ showed that enforcing the condition *α* + *β* = 1 led to a poor description of the experimental CVs for the one-electron reduction of 2-nitropropane, while relaxing this constraint (*α* + *β* ≠ 1) provided an excellent fit. Different literature studies also^56–60^ reported experimental systems where *α* + *β* ≠ 1. To our knowledge, no theoretical investigations have examined the influence of *α* + *β* ≠ 1 on cyclic voltammetry for metal electrodeposition. To address this gap, Fig. 3 demonstrates how variations in *α* + *β* affect the anodic peak. In this analysis, *α* is held constant at 0.5, while *β* takes values of 0.3, 0.5, and 0.7. The resulting deviation in the dimensionless anodic peak potential, observed when *α* + *β* ≠ 1 compared to the reference case of *α* + *β* = 1, is defined as Δ*η*_p_.

7 Δ*η*_*p*_ = *η*_*p*_*a*_(*α*+*β*≠1)_ − *η*_*p*_*a*_(*α*+*β*=1)_

8
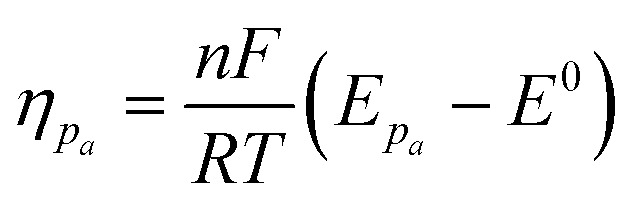


It is observed that the below eqn (9), used to evaluate the dimensionless peak-to-peak potential separation, Δ*Φ*_(*α*+*β*≠1)_, provides a better fit when the sum of the transfer coefficients deviates from unity. This parameter, Δ*Φ*_(*α*+*β*≠1)_, can be extracted from experimental cyclic voltammograms using eqn (1).

9 Δ*Φ*_(*α*+*β*≠1)_ = Δ*Φ*_(*α*+*β*=1)_ + Δ*η*_*p*_

Δ*η*_*p*_ depends on *α*, *β* and *ω*. In order to avoid interpolation equations excessively long, we define the following two types of Δ*η*_*p*_:Δ*η*_*p*_1__:Δ*η*_*p*_ when −3 ≤ log(*ω*) ≤ −1Δ*η*_*p*_2__:Δ*η*_*p*_ when log(*ω*) ≤ −3

**Fig. 4:**
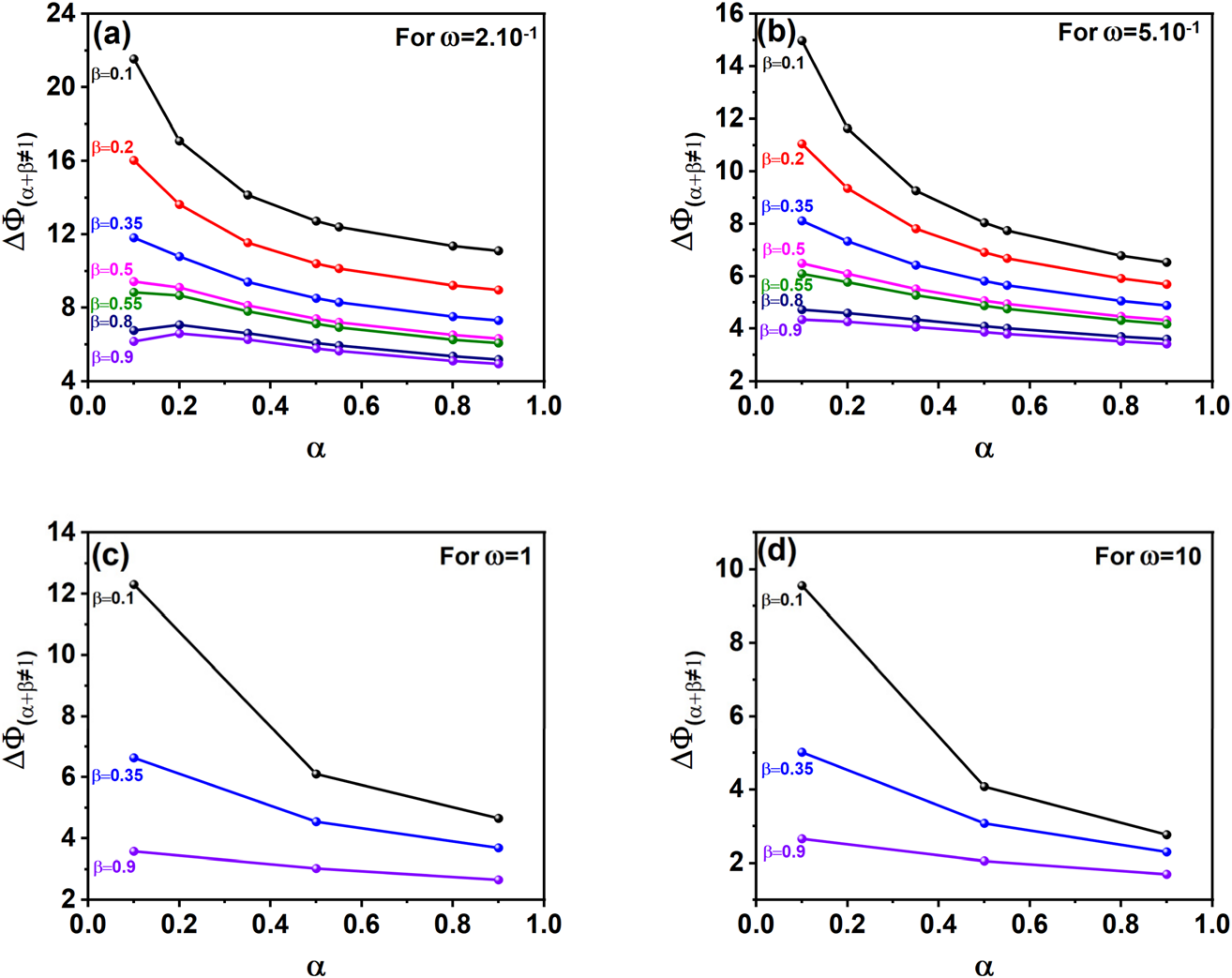
Kinetic curves presenting the effect of *α* and *β* (where *α* + *β* ≠ 1) and the kinetic rate constant *ω*, on the peak-to-peak potential separation (Δ*Φ*) for: (a) Δ*Φ*_(*α*+*β*≠1)_ for *ω* = 2 × 10^−1^, (b) Δ*Φ*_(*α*+*β*≠1)_ for *ω* = 5 × 10^−1^, (c) Δ*Φ*_(*α*+*β*≠1)_ for *ω* = 1, (d) Δ*Φ*_(*α*+*β*≠1)_ for *ω* = 10.

When analyzing a voltammogram characterized by specific values of *α*, *β*, and Δ*Φ*_(*α*+*β*≠1)_, an approximate estimation of the corresponding *ω* range can be made using the theoretical reference tools: the kinetic diagrams in Fig. 4 or the validation data provided in Table S1 (see SI). Once a given Δ*η*_*p*_1__, or Δ*η*_*p*_2__ is identified, its association with eqn (3) allows for the formulation of a new interpolation expression for the dimensionless peak-to-peak potential difference under conditions where *α* + *β* ≠ 1. Although interpolation equations cannot be derived when log(*ω*) > −1, it remains possible to construct new kinetic curves within this range.

Here below we present the development of equations permitting the calculation of the dimensionless standard heterogeneous rate constant for two different ranges:

#### 2.3.1. Interpolation equation for the dimensionless standard rate constant (*ω*): −3 ≤ log(*ω*) ≤ −1

In the range of −3 ≤ log(*ω*) ≤ −1, we obtained by interpolation the following eqn (10) for Δ*η*_*p*_1__.

10 Δ*η*_*p*_1__ = (*a* + *bα* + *cα*^2^) + (*a*_1_ + *b*_1_*α* + *c*_1_*α*^2^) × exp((*a*_2_ + *b*_2_*α* + *c*_2_*α*^2^) × *β*)

where:

11 *a* = 0.27594 + 1.43996 log *ω* − 0.01107 log *ω*^2^

12 *b* = −5.28228 − 9.8649 log *ω* + 0.04393 log *ω*^2^

13 *c* = 7.30133 + 19.49543 log *ω* − 0.31554 log *ω*^2^

14 *a*_1_ = −58.60695 − 88.52844 log *ω* − 12.87437 log *ω*^2^

15 *b*_1_ = 155.89195 + 214.46174 log *ω* + 53.23083 log *ω*^2^

16 *c*_1_ = −128.3385 − 175.8604 log *ω* − 43.75774 log *ω*^2^

17 *a*_2_ = −0.70606 + 4.20589 log *ω* + 0.80246 log *ω*^2^

18 *b*_2_ = −5.06505 − 5.5916 log *ω* − 1.31285 log *ω*^2^

19 *c*_2_ = 4.59224 + 4.88175 log *ω* + 1.12871 log *ω*^2^

*R*^2^ = 0.9981

log(*ω*) can be determined by substituting eqn (10) and (3) into eqn (9). This latter becomes:

20 Δ*Φ*_(*α*+*β*≠1)_ = Δ*Φ*_(*α*+*β*=1)_ + Δ*η*_*p*_1__

Although eqn (20) is lengthy and appears complex, it provides satisfactory results for the calculation of *k*^0^, in the range of −3 ≤ log(*ω*) ≤ −1, as demonstrated in both Theoretical and Experimental Validation Sections. Furthermore, in the experimental section, we found that eqn (34), which is simpler than eqn (20), also remains valid within an acceptable margin of error. Eqn (34) will be defined in the following section.

#### 2.3.2. Interpolation equation for the dimensionless standard rate constant (*ω*): log(*ω*) ≤ −3

In the case of an irreversible system, we developed hereinafter the eqn (33) which permitted the calculation of Δ*η*_*p*_2__ when log(*ω*) ≤ −3.

Fig. 3 shows typical electrochemical metal deposition voltammograms with same *α* and different *β*. Let consider Δ*η*_*p*_ between two curves. The first curve when *β* = *β*_1_ = 1 − *α*, and the second one when *β* = *β*_2_ ≠ 1 − *α* considered as the real experimental value.21
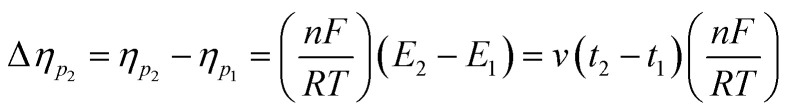


Once *t*_1_ (the anodic peak time of the curve when *α* + *β* = 1) is determined, Δ*η*_*p*_2__ can be calculated.

To determine *t*_1_ we proceeded as follows:

Given that the anodic electrical charges for the voltammograms are the same:

22 Qa_1_ = Qa_2_

Hence:23
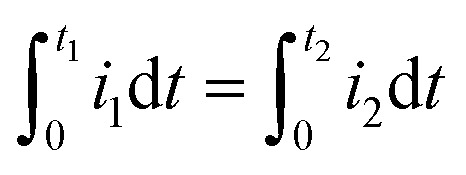
where:24
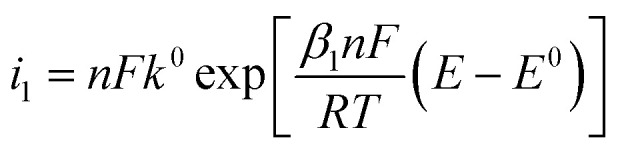
25
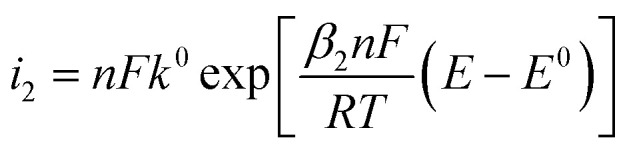


Since *E* = *vt*26
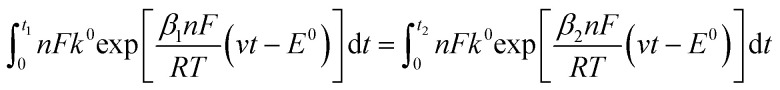
27
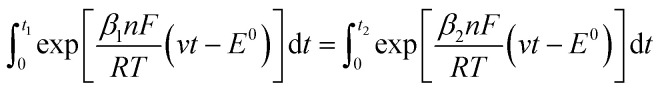
28
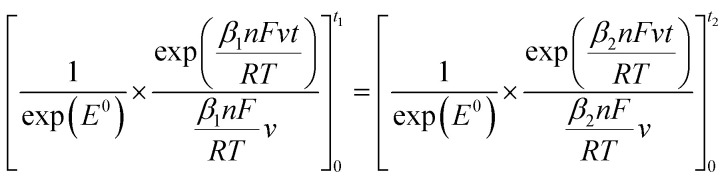
29
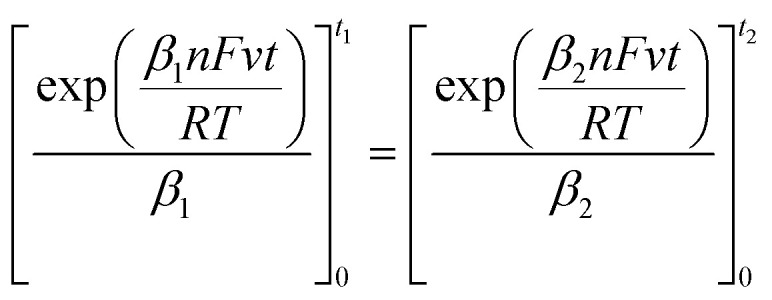
30
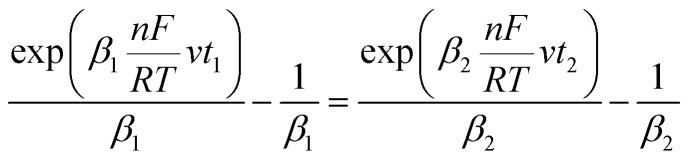
31
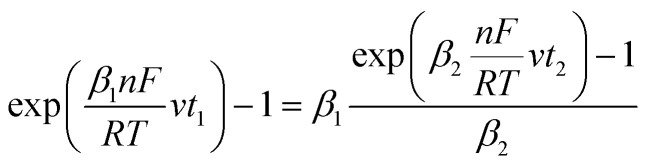
32
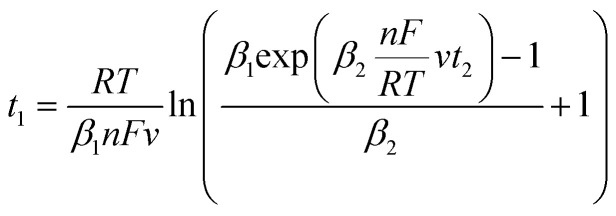


By replacing the eqn (32) in (21)

We obtain33
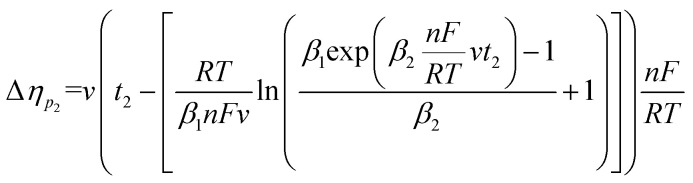


By determining *α* and *β*_2_ using Tafel plots, we can determine *β*_1_ = 1 − *α*. The parameter *t*_2_ represents the anodic peak time of the experimental curve. We can then calculate Δ*η*_*p*_2__ using eqn (33). Finally, by substituting eqn (33) and (3) into eqn (9). This latter becomes:

34 Δ*Φ*_(*α*+*β*≠1)_ = Δ*Φ*_(*α*+*β*=1)_ + Δ*η*_*p*_2__

#### 2.3.3. Kinetic curves for *α* + *β* ≠ 1 and when log(*ω*) > −1

As shown in Fig. 4, kinetic curves were generated for four representative values of log(*ω*) > −1. In this context, Fig. 4a–d provide practical reference curves for estimating *ω* values of 2 × 10^−1^, 5 × 10^−1^, 1, and 10, respectively. In this range, calculations are based exclusively on kinetic curves, as no interpolation equations have been developed. This reliance on direct curve analysis becomes even more critical when *α* + *β* ≠ 1, since each unique combination of *α* and *β* demands a specific kinetic curve for a given log(*ω*). As a result, the number of required curves becomes practically unlimited.

When Δ*Φ* is less than 25, the diagrams above can be used to estimate the dimensionless kinetic parameter (*ω*), provided that the values of *α*, *β*, and Δ*E*_p_ are known.

### 2.4. Theoretical validation of interpolation equations

Using MATLAB, we carried out a large set of simulations to test the validity of our interpolation equations across a broad range of electrochemical parameters. For each simulated cyclic voltammogram, values of *α*, *β*, and *ω* were varied. The peak-to-peak separation (Δ*E*_p_) was first extracted, then used to calculate a new estimated dimensionless kinetic parameter (denoted as *ω*′) *via* our interpolation equations. These calculated values were compared with the original input *ω*, and the results are compiled in Table S1. The MATLAB code used for these computations is included in the SI. Minor deviations between *ω* and *ω*′ values are attributed to approximation errors introduced during numerical computation.

The results in Table S1 show that the interpolation equations perform well across a wide range of *ω*, *α*, and *β* values, for both *α* + *β* = 1 and *α* + *β* ≠ 1 conditions. The accuracy of *k*^0^ estimation is highly sensitive to the value of *α* + *β*; assuming *α* = *β* = 0.5 or *α* + *β* = 1 without verification may lead to notable errors, particularly in irreversible systems.

For extreme values of *α*, *β* (<0.2 or >0.8), or when *α* + *β* is outside the typical range (<0.3 or >1.2), kinetic diagrams become less reliable. In such cases, interpolation equations offer more consistent results.

This work provides one of the most comprehensive validations of how *α*, *β*, and *α* + *β* affect Δ*E*_p_ and the accurate determination of *k*^0^ in metal electrodeposition reactions.

### 2.5. Experimental validation for silver, copper and rhenium ions reductions

The experimental cyclic voltammogram presented in Fig. S2 corresponds to the reduction of silver ions at a gold electrode, performed in HClO_4_ electrolyte with a scan rate of 50 mV s^−1^, the Fig. S3 corresponds to the reduction of copper ions at a platinum electrode in TEABF_4_ electrolyte at 100 mV s^−1^, while Fig. S4 represents the reduction of rhenium hexafluoride ReF_6_ at a vitreous carbon wire in LiF–NaF–KF eutectic mixture at a scan rate of 50 mV s^−1^. A sharp drop in the anodic peak is observed in all voltammograms, which is a characteristic feature of a metal electrodeposition reaction.

The reactions of these soluble–insoluble redox systems are as follows:

35 CF_3_SO_3_Ag + e^−^ ⇌ Ag + CF_3_SO_3_^−^

36 [Cu(CH_3_ CN)_4_]^+^ + e^−^ ⇌ Cu + 4CH_3_ CN

37 ReF_8_^2−^ + 6e^−^ ⇌ Re + 8F^−^

To determine *k*^0^, the anodic and cathodic charge transfer coefficients, along with the diffusion coefficient, are required (refer to the SI). For silver ions, Tafel analysis and the semi-integration method (see Fig. S2) yielded *α* = 0.302, *β* = 0.514, and *D* = 5.56 × 10^−10^ m^2^ s^−1^. For copper ions (see Fig. S3), the corresponding values were *α* = 0.727, *β* = 0.460, and *D* = 2.58 × 10^−9^ m^2^ s^−1^. For rhenium ions (see Fig. S4), the corresponding values were *α* = 0.130, *β* = 0.110, and *D* = 8 × 10^−10^ m^2^ s^−1^. The calculation of *k*^0^ is performed using both the kinetic diagrams and the interpolation equations developed in this work.

Based on the experimental data, the peak-to-peak separation (Δ*E*_p_) for silver ions, as shown in Fig. S2, is 0.12 V, calculated from Δ*E*_p_ = *E*_pa_ − *E*_pc_ = 0.07 − (−0.05). For copper ions, as shown in Fig. S3, is 0.23 V, calculated from Δ*E*_p_ = *E*_pa_ − *E*_pc_ = 0.08 − (−0.16). In comparison, the rhenium ions cyclic voltammogram in Fig. S4 gives a Δ*E*_p_ of 1.17 V, determined as Δ*E*_p_ = *E*_pa_ − *E*_pc_ = 3.43 − 2.26. Using eqn (1), these values yield the dimensionless peak-to-peak potential separations: Δ*Φ*_1_ for silver, Δ*Φ*_2_ for copper and Δ*Φ*_3_ for rhenium:38
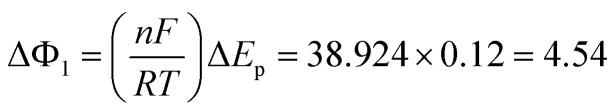
39
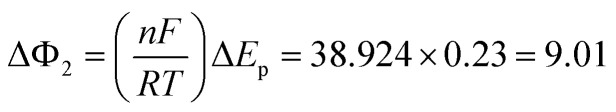
40
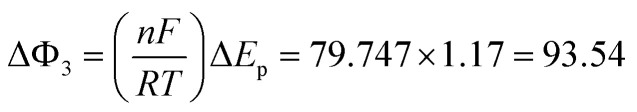


Standard heterogeneous rate constant (*k*^0^) values for the reduction of silver ions, copper ions as well as of rhenium ions, obtained from different kinetic curves and calculated from different equations are obtained in Tables 1–3, respectively.

**Table 1:** Dimensionless rate constant (*ω*) and standard heterogeneous rate constants (*k*^0^) values for the reduction of silver ions

Calculation methods of *k*^0^	*ω*	*k* ^0^, 10^−6^ m s^−1^
Kinetic curves where *α* + *β* = 1 (Fig. 2)	≈0.81	≈11.75
Kinetic curves where *α* + *β* ≠ 1 (Fig. 4)	≈1	≈14.51

**Table 2:** Dimensionless rate constant (*ω*) and standard heterogeneous rate constants (*k*^0^) values for the reduction of copper ions

Calculation methods of *k*^0^	*ω*, 10^−2^	*k* ^0^, 10^−7^ m s^−1^
Kinetic curves where *α* + *β* ≠ 1 (Fig. 4)	≈20	≈12.49
Interpolation equation where *α* + *β* = 1 (3)	14.42	9.0
Interpolation equation where *α* + *β* ≠ 1 (20)	9.58	5.98
Interpolation equation where *α* + *β* ≠ 1 (34)	8.54	5.33

**Table 3:** Dimensionless rate constant (*ω*) standard heterogeneous rate constant (*k*^0^) values for the reduction of rhenium ions

Calculation methods of *k*^0^	*ω*, 10^−4^	*k* ^0^, 10^−8^ m s^−1^
Kinetic curves where *α* + *β* = 1 (Fig. 2)	≈0.46	≈0.29
Interpolation equation where *α* + *β* = 1 (3)	0.13	0.08
Interpolation equation where *α* + *β* ≠ 1 (20)	17	10.59
Interpolation equation where *α* + *β* ≠ 1 (34)	42	21.81

For silver ions, the dimensionless kinetic parameter *ω* can be estimated using two different approaches. When assuming *α* + *β* = 1, the value is derived from the kinetic diagram in Fig. 2b using a proportional scale: *ω* ≈ (1.8 cm × 0.9)/2 cm = 0.81. Alternatively, under the condition *α* + *β* ≠ 1, Fig. 4c suggests a value of *ω* close to 1. With *ω* and *α* known, the standard heterogeneous rate constant *k*^0^ is computed using eqn (S14), yielding approximately 11.75 × 10^−6^ m s^−1^ (for *ω* ≈ 0.81) and 14.51 × 10^−6^ m s^−1^ (for *ω* ≈ 1).

All the interpolation equations, whether for *α* + *β* = 1 or *α* + *β* ≠ 1, are valid for *ω* ≤ 10^−1^, whereas the estimated *ω* values for silver ions are 0.81 and approximately 1. Hence, these values are outside the applicable range.

For copper ions, *ω* was estimated using the kinetic diagram in Fig. 4a (*α* + *β* ≠ 1) suggesting a value of *ω* close to 0.2. With this *ω* value and the known charge transfer coefficient *α*, the standard rate constant *k*^0^ was calculated using eqn (S14), resulting in *k*^0^ ≈ 12.49 × 10^−7^ m s^−1^.

Using the interpolation equations for the three cases, *α* + *β* = 1 (eqn (3)), *α* + *β* ≠ 1 (eqn (20)), and *α* + *β* ≠ 1 for irreversible systems (eqn (34)), the calculated *k*^0^ value for copper ions are 9.0 × 10^−7^ m s^−1^, 5.98 × 10^−7^ m s^−1^, and 5.33 × 10^−7^ m s^−1^, respectively.

For rhenium ions, the dimensionless kinetic parameter *ω* was estimated using the kinetic diagram in Fig. 2b (*α* + *β* = 1). Applying the rule of three gives *ω* ≈ (3.7 cm × 0.00009)/7.2 cm = 0.46 × 10^−4^. With this *ω* value and the known charge transfer coefficient *α*, the standard rate constant *k*^0^ was calculated using eqn (S14), resulting in *k*^0^ ≈ 0.29 × 10^−8^ m s^−1^.

Using the interpolation equations for the three cases, *α* + *β* = 1 (eqn (3)), *α* + *β* ≠ 1 (eqn (20)), and *α* + *β* ≠ 1 in irreversible systems (eqn (34)), the calculated standard rate constants *k*^0^ for rhenium ions are 0.08 × 10^−8^ m s^−1^, 10.59 × 10^−8^ m s^−1^, and 21.81 × 10^−8^ m s^−1^, respectively. Since eqn (34) provides a simpler approach and yields a comparable result, it may be used in place of eqn (20) for practical *k*^0^ calculation.

**Fig. 5:**
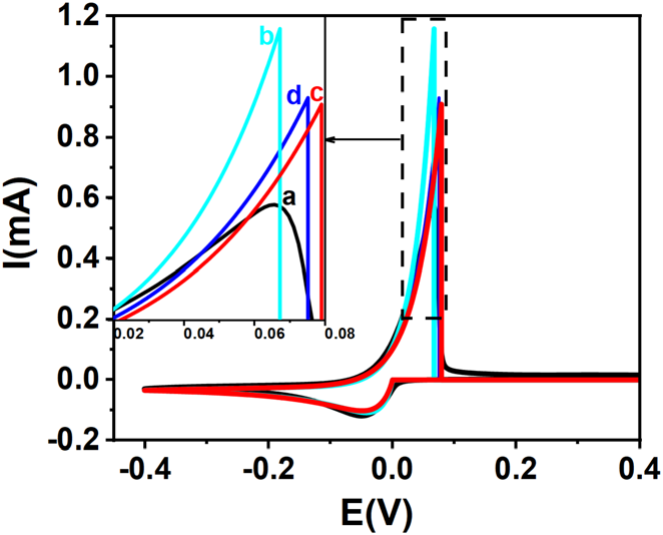
Experimental and theoretical cyclic voltammograms for Ag^+^/Ag redox couple: (a) experimental CV (black solid line) and (b–d) theoretical curves: (b) cyan solid line, from kinetic curves *α*+*β* = 1 (Fig. 2): *k*^0^ = 11.75 × 10^−6^ m s^−1^, *α* = 0.302, *β* = 0.698, (*α* + *β* = 1); (c) red solid line, from kinetic curves *α* + *β* = 1 (Fig. 2): *k*^0^ = 11.75 × 10^−6^ m s^−1^, *α* = 0.302, *β* = 0.514 (*α* + *β* ≠ 1); (d) blue solid line, from kinetic curves *α* + *β* ≠ 1 (Fig. 4): *k*^0^ = 14.51 × 10^−6^ m s^−1^, *α* = 0.302, *β* = 0.514 (*α* + *β* ≠ 1). Inset: magnified view of the anodic peaks.

Next, we simulate the cyclic voltammograms corresponding to the *k*^0^ values determined for silver, copper and rhenium ions. Fig. 5 presents both the simulated and experimental cyclic voltammograms for silver ion reduction. The simulations were carried out using the previously determined values of the charge transfer coefficient *α*, the diffusion coefficient *D*, and the standard rate constant *k*^0^. For the anodic transfer coefficient *β*, two approaches were considered: the value obtained from Tafel analysis and the one calculated using the relation *β* = 1 − *α*.

Fig. 5 compares experimental and simulated cyclic voltammograms for silver ion reduction. In Fig. 5b, the simulated curve generated using *k*^0^ = 11.75 × 10^−6^ m s^−1^, with *α* = 0.302 and *β* estimated as 1 − *α* = 0.698 (assuming *α* + *β* = 1), shows noticeable deviation from the experimental voltammogram (Fig. 5a), particularly near the anodic peak. Conversely, Fig. 5c displays a much better fit when the same *k*^0^ and *α* values are used, but *β* is taken from the Tafel plot (*β* = 0.514), highlighting the importance of using experimentally derived *β* values rather than assuming *β* = 1 − *α*.^52^ Finally, Fig. 5d shows the simulated curve using *k*^0^ = 14.51 × 10^−6^ m s^−1^, obtained from the kinetic model where *α* + *β* ≠ 1, which provides the closest agreement with the experimental data.

**Fig. 6:**
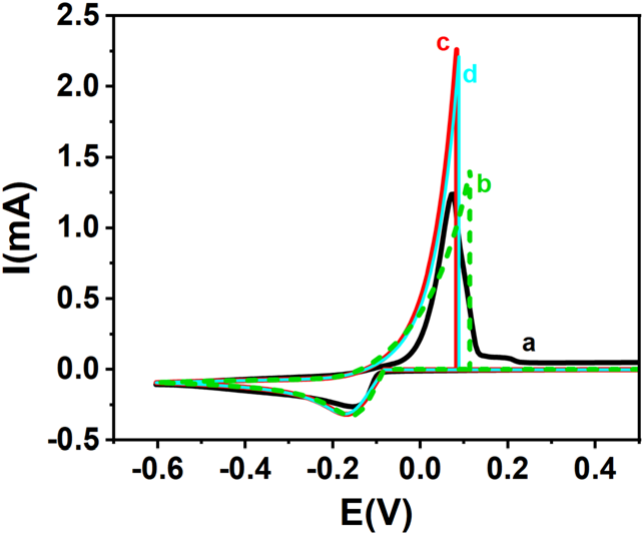
Experimental and theoretical cyclic voltammograms for Cu^+^/Cu redox couple: (a) experimental CV (black solid line), along with (b–d) theoretical curves derived from various interpolation equations: (b) green dashed line: theoretical curve from eqn (3), with *k*^0^ = 9.0 × 10^−7^ m s^−1^, *α* = 0.727, *β* = 0.273 (*α* + *β* = 1); (c) red solid line: eqn (20), *k*^0^ = 5.98 × 10^−7^ m s^−1^, *α* = 0.727, *β* = 0.460 (*α* + *β* ≠ 1); (d) cyan solid line: eqn (34), *k*^0^ = 5.33 × 10^−7^ m s^−1^, *α* = 0.727, *β* = 0.460 (*α* + *β* ≠ 1).

Fig. 6 represents simulated and experimental cyclic voltammograms for copper ions using various *k*^0^ values derived from different interpolation equations. When *k*^0^ = 9.0 × 10^−7^ m s^−1^, obtained from the interpolation equation assuming *α* + *β* = 1 (eqn (3)), the resulting curves (Fig. 6b), assuming *α* + *β* = 1, shows deviations from the experimental voltammogram (Fig. 6a). In contrast, the simulation shown in Fig. 6c and d, based on *k*^0^ = 5.98 × 10^−7^ m s^−1^ and *k*^0^ = 5.33 × 10^−7^ m s^−1^ obtained from eqn (20) and eqn (34) where *α* + *β* ≠ 1, aligns much more closely with the experimental data.

**Fig. 7:**
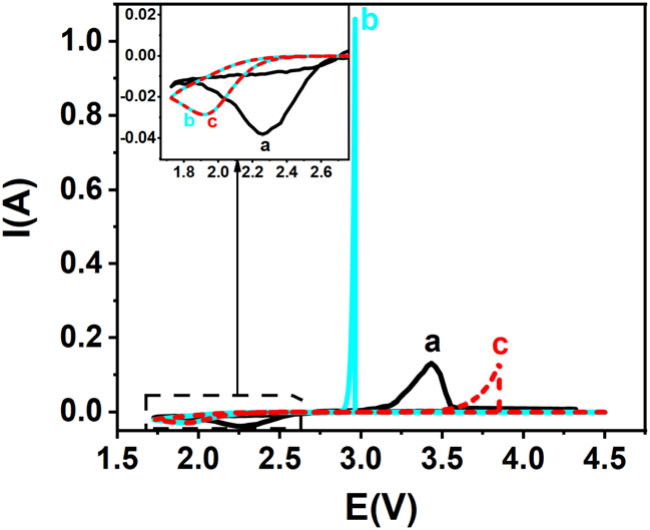
Experimental and theoretical cyclic voltammograms for Re^6+^/Re redox couple: (a) experimental CV (black solid line), adapted from Affoune *et al.*, *J. Appl. Electrochem.*, 2002, 32, 721–728, https://doi.org/10.1023/A:1016532912889, with permission from Springer Nature; (b and c) theoretical curves are shown for comparison: (b) cyan solid line: *α* = 0.130, *β* = 0.870 (*α* + *β* = 1); (c) red dashed line: *α* = 0.130, *β* = 0.110 (*α* + *β* ≠ 1). The standard rate constant was calculated from the kinetic curves *α* + *β* = 1 (Fig. 2): *k*^0^ = 0.29 × 10^−8^ m s^−1^. Inset: magnified view of the cathodic peaks.

**Fig. 8:**
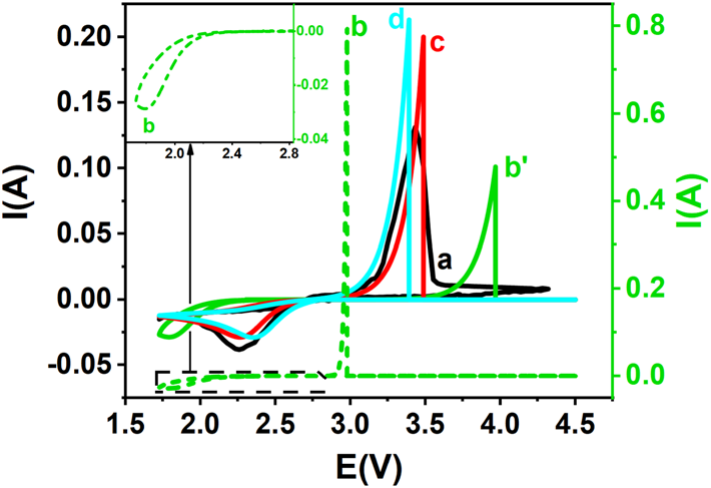
Experimental and theoretical cyclic voltammograms for Re^6+^/Re redox couple: (a) experimental CV (black solid line), adapted from Affoune *et al.*, *J. Appl. Electrochem.*, 2002, 32, 721–728, https://doi.org/10.1023/A:1016532912889, with permission from Springer Nature, along with (b–d) theoretical curves derived from various interpolation equations: (b) green dashed line (right *Y*-axis): theoretical curve from eqn (3), with *k*^0^ = 0.08 × 10^−8^ m s^−1^, *α* = 0.130, *β* = 0.870 (*α*+*β* = 1); (b′) green solid line (left *Y*-axis): eqn (3), *k*^0^ = 0.08 × 10^−8^ m s^−1^, *α* = 0.130, *β* = 0.110 (*α* + *β* ≠ 1); (c) red solid line (left *Y*-axis): eqn (20), *k*^0^ = 10.59 × 10^−8^ m s^−1^, *α* = 0.130, *β* = 0.110 (*α* + *β* ≠ 1); (d) cyan solid line (left *Y*-axis): eqn (34), *k*^0^ = 21.81 × 10^−8^ m s^−1^, *α* = 0.130, *β* = 0.110 (*α* + *β* ≠ 1). Inset: zoomed view of the cathodic peak (b).

Fig. 7 and Fig. 8 display the experimental and simulated cyclic voltammograms for rhenium ion reduction. The simulations were performed using the previously determined parameters: *α*, the diffusion coefficient *D*, and the standard rate constant *k*^0^. For the anodic charge transfer coefficient *β*, two approaches were considered, either the value obtained directly from Tafel analysis or the one estimated using the assumption *β* = 1 − *α*.

As shown in Fig. 7, the simulated voltammogram in Fig. 7b, calculated using *k*^0^ = 0.29 × 10^−8^ m s^−1^ from the kinetic curves assuming *α* + *β* = 1 (Fig. 2) (with *α* = 0.130 and *β* = 1 − *α* = 0.870), deviates significantly from the experimental data (Fig. 7a), particularly at the anodic peak. In Fig. 7c, a second simulation using the same *k*^0^ and *α* values but with *β* obtained from the Tafel analysis (*β* = 0.110) shows a partial improvement where the anodic peak current is more aligned with the experimental value. However, the overall shape remains inconsistent. These discrepancies confirm that the kinetic curves assuming *α* + *β* = 1 (Fig. 2) are not appropriate in this case, as the experimentally determined sum *α* + *β* is approximately 0.24.

Fig. 8 compares simulated and experimental cyclic voltammograms for rhenium ions using various *k*^0^ values derived from different interpolation equations. When *k*^0^ = 0.08 × 10^−8^ m s^−1^, obtained from the interpolation equation assuming *α* + *β* = 1 (eqn (3)), the resulting curves (Fig. 8b and b′), whether assuming *α* + *β* = 1 or ≠ 1, show significant deviations from the experimental voltammogram (Fig. 8a). In contrast, the simulation shown in Fig. 8c, based on *k*^0^ = 10.59 × 10^−8^ m s^−1^ obtained from eqn (20) (*α* + *β* ≠ 1), aligns much more closely with the experimental data. Additionally, Fig. 8d presents the simulation using *k*^0^ = 21.81 × 10^−8^ m s^−1^, calculated from the interpolation equation for irreversible systems (eqn (34)). Overall, the simulated voltammograms using interpolation equations tailored for *α* + *β* ≠ 1, particularly eqn (20), show the best agreement with experimental results.

These results suggest that the number of electrons involved in the redox process may significantly affect the estimated values of *k*^0^. In particular, the one-electron transfer of silver leads to a comparatively high *k*^0^ (14.51 × 10^−6^ m s^−1^), whereas the multielectron transfer of rhenium results in a much smaller value (10.59 × 10^−8^ m s^−1^). Such differences are consistent with the general trend that multielectron processes are kinetically less favorable, as they require more complex reorganization steps at the electrode–electrolyte interface.

It is generally assumed that *α* + *β* = 1, so that when one coefficient is determined, the other can be deduced.^61^ In electrodeposition studies, authors typically determine only the cathodic charge transfer coefficient, which is directly involved in the electrodeposition reaction.^62,63^ The anodic charge transfer coefficient is usually determined in studies focused on the oxidation or corrosion of metallic substrates.^64,65^ However, the literature shows that for soluble–soluble systems,^56,57,66,67^ there are significantly more references where the sum of (*α* + *β*) differs from unity in a single article, compared to electrodeposition systems.^53,58,59^ The results we obtained indicate that the sum (*α* + *β*) varies across different cases. For rhenium, (*α* + *β*) is 0.24; for silver, it is 0.816; and for copper, it is 1.187. Through this work, we have demonstrated that assuming *α* + *β* = 1 can lead to significant errors, as *k*^0^ is highly sensitive to the individual values of *α* and *β*.

The simulated voltammograms exhibit a higher peak current than the experimental ones because the simulated curve reaches a maximum current and then drops sharply, unlike the smoother behavior of the experimental curve. Despite this small discrepancy, the methodology we applied here for silver, copper and rhenium provided a reliable determination of *k*^0^.

## 3. Experimental and computational methods

### 3.1. Theory

The theoretical approach used to simulate cyclic voltammograms for the metal electrodeposition reactions is described in detail in the SI file.

### 3.2. Materials and methods

#### 3.2.1. Calculation methods

The numerical simulations were carried out using Fortran 90 and compiled with Microsoft Fortran PowerStation 4.0. Post-processing and graphical analysis of the simulation data were performed using Origin 2018, which offered a complete suite of tools for data visualization. Using the non-linear fitting tools of Origin software, we carefully analysed our kinetic curves (Fig. 2) to derive the equations. For Δ*ϕ* = *f*(*α*), we applied the Rational/Holliday function with the Levenberg–Marquardt algorithm, while for Δ*ϕ* = *f*(*ω*) we used Exponential models (ExpDec1, Exp2PMod1, Exp2P) with the same algorithm.

The charge transfer coefficient was estimated through Tafel plot analysis, while the diffusion coefficient was evaluated using the semi-integration technique, based on the method originally proposed by Oldham.^16,68,69^

#### 3.2.2. Reagents

For the electrochemical reduction reaction of silver ions, we used silver trifluoromethanesulfonate CF_3_SO_3_Ag (10 mM) in perchloric acid HClO_4_ (1 M) from Sigma-Aldrich Company. For the electrochemical reduction reaction of copper ions, we used tetrakis(acetonitrile)copper(i) tetrafluoroborate [Cu(CH_3_CN)_4_]BF_4_ (10 mM) from (TCI) in tetraethylammonuim tetrafluoroborate TEABF_4_ (0.1 M) from (ABCR). For the electrochemical reduction reaction of rhenium ions, we used rhenium hexafluoride ReF_6_ (26 mM) in LiF–NaF–KF eutectic mixture from Sigma-Aldrich Company; more details on the preparation of rhenium electrolyte are provided in the article of Affoune *et al.*^18^ All reagents were used without purification.

#### 3.2.3. Instrumentation and procedures

The cyclic voltammetric measurements for the reductions of CF_3_SO_3_Ag and [Cu(CH_3_CN)_4_]BF_4_ were carried out using an Autolab model PGSTAT302N potentiostat and for the reduction of ReF_6_ a potentiostat–galvonostat (PAR EG&G model 273), respectively.

For the reduction of CF_3_SO_3_Ag, a gold disc electrode (0.0707 cm^2^) was employed, with platinum wire as a counter electrode and a silver wire as a comparison electrode. For the reduction of [Cu(CH_3_CN)_4_]BF_4_, a platinum electrode (0.0707 cm^2^) was employed, with platinum wire as a counter electrode and a copper wire as a comparison electrode. The reduction of ReF_6_ was performed using a vitreous carbon wire (1.884 cm^2^) as a working electrode, graphite crucible as a counter electrode, and a platinum wire as a comparison electrode. The potentials were referred to the equilibrium potential of K^+^/K couple, the cathodic limit of the solvent.

The potential was swept between 0.4 V and −0.4 V at a scan rate of 50 mV s^−1^ for silver ions reduction, 0.7 V and −0.6 V at a scan rate of 100 mV s^−1^ for copper ions reduction and between 3.73 V and 1.73 V at a scan rate of 50 mV s^−1^ for rhenium reduction. The first two reactions were carried out at room temperature, whereas the rhenium ions reduction was performed at 600 °C.

## 4. Conclusions

Using the Butler–Volmer framework and a semi-analytical method, cyclic voltammograms were simulated for metal electrodeposition across reversible, quasi-reversible, and irreversible regimes. Kinetic curves were constructed from peak-to-peak separations over a wide range of *α* and dimensionless rate constants (*ω*), leading to interpolation equations for both *α* + *β* = 1 and *α* + *β* ≠ 1.

Experimental validation was performed *via* silver, copper and rhenium ions reduction. The extracted parameters were:

Silver couple: *α* = 0.302, *β* = 0.514, *D* = 5.56 × 10^−10^ m^2^ s^−1^, *k*^0^ = 14.51 × 10^−6^ m s.^−1^

Copper couple: *α* = 0.727, *β* = 0.460, *D* = 2.58 × 10^−9^ m^2^ s^−1^, *k*^0^ = 5.98 × 10^−7^ m s.^−1^

Rhenium couple: *α* = 0.130, *β* = 0.110, *D* = 8 × 10^−10^ m^2^ s^−1^, *k*^0^ = 10.59 × 10^−8^ m s.^−1^

Simulated voltammograms closely matched the experimental data, confirming the reliability of the proposed method.

This study provides a validated and practical approach for determining the standard rate constant *k*^0^ in metal electrodeposition systems using both kinetic curves and interpolation equations. Experimental results show that the derived *k*^0^ values reproduce cyclic voltammograms with excellent agreement. The interpolation equations offer superior precision, especially when *α* + *β* deviates from 1; where traditional kinetic curves become unreliable.

Importantly, our findings underline that assuming *α* + *β* = 1 can lead to significant errors, as *k*^0^ is highly sensitive to the individual values of *α* and *β*. This work delivers a generalizable method for extracting *k*^0^ from CV data, supported by both theory and experiment.

To our knowledge, it is the most thorough investigation to date on how *α*, *β*, and their sum influence cyclic voltammetry and peak-to-peak separation, filling key gaps in previous models and offering tools of wide relevance in electrochemical kinetics.

## Author contributions

Rania Saad Guermeche: data curation, formal analysis, software, visualization, writing – original draft, writing – review & editing. Abed Mohamed Affoune: conceptualization, data curation, formal analysis, methodology, investigation, project administration, supervision, validation, visualization, writing – original draft, writing – review & editing. Sabrina Houam: data curation, formal analysis, validation, writing – review & editing. Imene Atek: data curation, formal analysis, validation, writing – review & editing. Christine Vautrin-Ul: formal analysis, writing – review & editing. Mouna Nacef: software, writing – review & editing. Mohamed Lyamine Chelaghmia: validation, writing – review & editing. Hubert H. Girault: resources, validation, visualization, writing – review & editing. Craig E. Banks: validation, visualization, writing – review & editing. Ilhem Djaghout: supervision, writing – review & editing. Jacques Bouteillon: investigation (posthumous contribution of previously published experimental data used in this study). Jean Claude Poignet: investigation (posthumous contribution of previously published experimental data used in this study).

## Conflicts of interest

There are no conflicts to declare.

## Supplementary Material

SC-016-D5SC05636E-s001

SC-016-D5SC05636E-s002

SC-016-D5SC05636E-s003

SC-016-D5SC05636E-s004

SC-016-D5SC05636E-s005

SC-016-D5SC05636E-s006

SC-016-D5SC05636E-s007

SC-016-D5SC05636E-s008

SC-016-D5SC05636E-s009

## Data Availability

The data supporting this article have been included as part of the supplementary information (SI). Supplementary information: computational details of cyclic voltammetry simulation, effect of switching potential, analysis of charge transfer and diffusion coefficients, theoretical validation of interpolation equations, MATLAB code used for kinetic calculations, nomenclature and additional references. See DOI: https://doi.org/10.1039/d5sc05636e.
