# Supplementary figures and images for: Exploring the determination of the standard rate constant in electrochemical metal deposition: theory and experiment
Source: Chem Sci. 2025 Oct 8;16(45):21562–72. doi: 10.1039/d5sc05636e (PMC12529109; doi:10.1039/d5sc05636e)

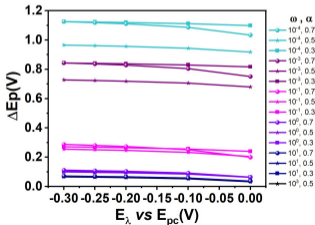

Supplement: SC-016-D5SC05636E-s002 [file SC-016-D5SC05636E-s002.pdf]

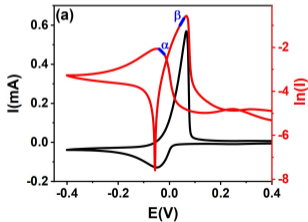

Supplement: SC-016-D5SC05636E-s003 [file SC-016-D5SC05636E-s003.pdf]

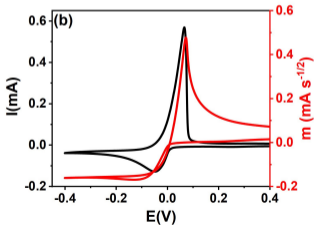

Supplement: SC-016-D5SC05636E-s004 [file SC-016-D5SC05636E-s004.pdf]

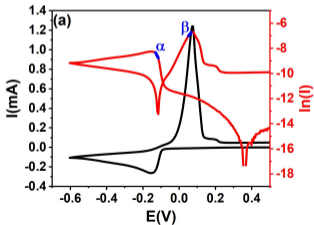

Supplement: SC-016-D5SC05636E-s005 [file SC-016-D5SC05636E-s005.pdf]

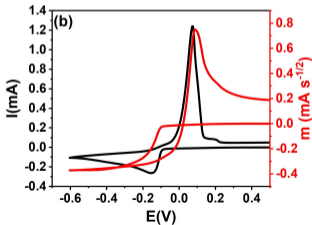

Supplement: SC-016-D5SC05636E-s006 [file SC-016-D5SC05636E-s006.pdf]

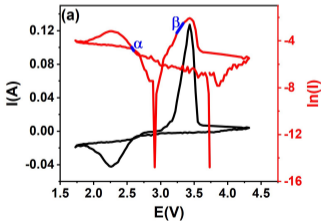

Supplement: SC-016-D5SC05636E-s007 [file SC-016-D5SC05636E-s007.pdf]

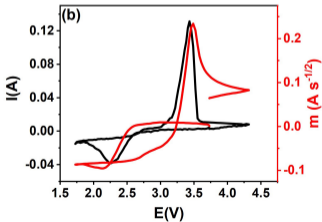

Supplement: SC-016-D5SC05636E-s008 [file SC-016-D5SC05636E-s008.pdf]
