# Supplementary material for: Exploring the determination of the standard rate constant in electrochemical metal deposition: theory and experiment
Source: Chem Sci. 2025 Oct 8;16(45):21562–72. doi: 10.1039/d5sc05636e (PMC12529109; doi:10.1039/d5sc05636e)
Supplement: SC-016-D5SC05636E-s009 [file SC-016-D5SC05636E-s009.pdf]

Supplementary Information (SI) for Chemical Science.
This journal is © The Royal Society of Chemistry 2025

# Supporting Information

## Exploring the Determination of the Standard Rate Constant in Electrochemical Metal Deposition: Theory and Experiment

*Rania Saad Guermeche [a], Abed Mohamed Affoune [a]*, Sabrina Houam [a], Imene Atek [b], Christine Vautrin-Ul [c], Mouna Nacef [a], Mohamed Lyamine Chelaghmia [a], Hubert H. Girault [d], Craig E. Banks [e], Ilhem Djaghout [a], Jacques Bouteillon [f]**, and Jean Claude Poignet [f]***

[a] Laboratory of Industrial Analysis and Material Engineering, Department of Process Engineering, University 8 May 1945 Guelma, BP 401, Guelma 24000, Algeria

[b] Laboratory of Process Engineering for Sustainable Development and Health Products, Preparatory Classes Department, National Polytechnic School of Constantine, Constantine 25000, Algeria

[c] Laboratoire ICMN Interfaces, Confinement, Matériaux et Nanostructures, UMR7374, Université d'Orléans–CNRS, 1b rue de la Férollerie, 45071, Orléans Cedex 2, France

[d] Laboratoire d'Electrochimie Physique et Analytique, École Polytechnique Fédérale de Lausanne, EPFL Valais Wallis, Rue de l'Industrie 17, Case Postale 440, CH-1951Sion, Switzerland

[e] Faculty of Science and Engineering, Manchester Metropolitan University, Dalton Building, Chester Street, Manchester M1 5GD, Great Britain

[f] Laboratoire d'électrochimie et de physicochimie des matériaux et des interfaces, 1130 rue de la Piscine, 38402 Saint Martin d'Hères, France

* E-mail:affoune2@gmail.com

** We gratefully acknowledge the late Prof. Jacques Bouteillon and the late Prof. Jean-Claude Poignet for their valuable contributions to specific aspects of this work

## Table of Contents

**I Cyclic Voltammetry Computation** **S3**

**II Switching Potential ($E_\lambda$) Effect** **S4**

**III Charge Transfer Coefficients and Diffusion Coefficients Determination of Silver, Copper and Rhenium Ions Reductions** **S5**

**IV Theoretical Validation of Interpolation Equations** **S7**

**V MATLAB Code for log($\omega$) Interpolation Equations Calculation** **S8**

**Nomenclature** **S10**

**References** **S11**

## I Cyclic Voltammetry Computation

A semi-analytical approach inspired by Nicholson's method was employed to simulate cyclic voltammograms for the reduction of soluble metal ions ($M^{n+}$) into a solid metal (M). The process corresponds to the electrodeposition reaction represented by the following equation:

$$M^{n+} + n\acute{e} \Leftrightarrow M \quad \text{(S1)}$$

The variation in current is governed by the Butler–Volmer equation, which relates the electrode kinetics to the overpotential and charge transfer parameters:

$$I = nFAk^0 \left[ C_M(0,t) exp\left(\frac{\beta nF(E-E^0)}{RT}\right) - C_{M^{n+}}(0,t) exp\left(\frac{-\alpha nF(E-E^0)}{RT}\right)\right] \quad \text{(S2)}$$

Under the assumption that both migration and convection are negligible, species transport in the electrolyte is described by Fick's second law:

$$\frac{\partial C(x,t)}{\partial t} = D\frac{\partial^2 C(x,t)}{\partial x^2} \quad \text{(S3)}$$

In cyclic voltammetry (CV), the potential E(t) is varied over time according to the following relations:

$$0 < t \leq \lambda \;,\; E(t) = E_i - vt \quad \text{(S4)}$$
$$t > \lambda \;,\; E(t) = E_i - 2v\lambda + vt \quad \text{(S5)}$$

where:

$E_i$ : initial potential
$\lambda$ : switching time

The initial potential ($E_i$) obeys the Nernst equation:

$$E_i = E_{eq} = E^0 + \frac{RT}{nF} ln\left(\frac{C^*_{M^{n+}}}{C^*_M}\right) \quad \text{(S6)}$$

Considering the following initial and boundary conditions, and assuming that only the oxidized specie ($M^{n+}$) is initially present in the bulk solution:

$$t = 0\,, x \geq 0, C_{M^{n+}}(x,0) = C^*_{M^{n+}} \quad \text{(S7)}$$
$$t > 0, x \to \infty, C_{M^{n+}}(\infty,t) = C^*_{M^{n+}} \quad \text{(S8)}$$
$$t > 0, x \to 0\,, J_{M^{n+}}(0,t) = \frac{I(t)}{nFA} = -D_{M^{n+}}\left[\frac{\partial C_{M^{n+}}(x,t)}{\partial x}\right]_{x=0} \quad \text{(S9)}$$

By applying the Laplace transform to solve Fick's second law (S3) under the specified initial and boundary conditions (S7-S9), an analytical expression is derived for the concentration of $M^{n+}$ species at the surface of electrode (x=0) at any time (t), $C_{M^{n+}}(0,t)$, is obtained:

$$C_{M^{n+}}(0,t) = C^*_{M^{n+}} + \frac{1}{nFA\sqrt{\pi D_{M^{n+}}}}\int_0^t \frac{I(\tau)}{\sqrt{t-\tau}} d\tau \quad \text{(S10)}$$

Concerning the metallic specie:[1]

$$C_M(0,t) = 1 \quad \text{(S11)}$$

Combining equations S2, S4-S6, S10-S11, the expression of the current can be given as follows:

$$I(t) = nFA\, C^{*}_{M^{n+}} D^{1/2}_{M^{n+}} \left(\frac{nF}{RT}\right)^{1/2} v^{1/2} \pi^{1/2} \chi(\sigma t) \tag{S12}$$

Where the dimensionless current $\chi(\sigma t)$ is given by the integral:

$$\int_0^{\sigma t} \frac{\chi(z)}{\sqrt{\sigma t - z}} dz = -\left[-1 - \frac{1}{\omega}\chi(\sigma t)[S(\sigma t)]^{\alpha} + [S(\sigma t)]^{(\alpha+\beta)}\right] \tag{S13}$$

The reversibility factor ω was described by Krulic :[2]

$$\omega = \frac{k^0}{\theta^{\alpha}\sqrt{\pi \sigma D_{M^{n+}}}} \tag{S14}$$

where:

$$\sigma = \frac{nFv}{RT} \tag{S15}$$

and

$$\theta = \exp\left(\left(\frac{nF}{RT}\right)[E_i - E^0]\right) = \frac{C^{*}_{M^{n+}}}{C^0} \tag{S16}$$

where $C^0$ is the standard concentration of 1 mol $L^{-1}$.

To make it easier to model, the dimensioned variables are transformed to dimensionless form, where:

$$S(\sigma t) = exp(-\sigma t) \tag{S17}$$

and where the initial potential is expressed as:

$$Init = \frac{nF}{RT}(E_i - E^0) \tag{S18}$$

and the applied potential as:

$$\Phi = \frac{nF}{RT}(E(t) - E^0) = Init - \sigma t \tag{S19}$$

The numerical method developed by Nicholson[3] was used to calculate the integral (S13), giving the following algorithms:

$$\chi(1)\sqrt{K} + \sum_{i=1}^{K-1} \sqrt{K-i}[\chi(i+1) - \chi(i)] = -\frac{1}{2\sqrt{\delta}}\left[-1 - \chi(\delta K)\omega[S(\delta K)]^{\alpha} + [S(\delta K)]^{(\alpha+\beta)}\right] \tag{S20}$$

*K=1, 2, 3 …N*

where (δ) represents the calculation step.

Providing the values of the δ, Init, Φ, α, β, ω in the corresponding algorithm, the dimensionless cyclic voltammograms can be computed.

## II Switching Potential ($E_\lambda$) Effect

Figure S1 illustrates the impact of switching potential on $\Delta E_p$, evaluated at five different values (0V, -0.1V, -0.2V, -0.25V and -0.3V) relative to the cathodic peak potential. Simulations were performed across reversible, quasi-reversible, and irreversible regimes. For each case, cathodic transfer coefficients of 0.3, 0.5, and 0.7 were used under the condition α+β=1. The results indicate that, in reversible systems, $\Delta E_p$

remains constant regardless of the value of α, highlighting its independence from the charge transfer coefficient in this regime.

As highlighted in Figure S1, a slight variation in $\Delta E_p$ appears when $E_\lambda$>-0.2V vs $E_{pc}$. Notably, this behavior persists even when the condition $\alpha + \beta \neq 1$.

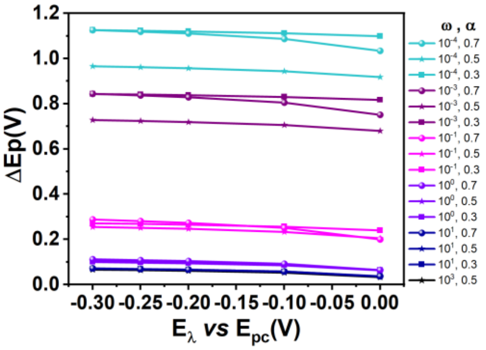


**Figure S1.** Presentation of $\Delta E_p$ as a function of $E_\lambda$, ω and α

## III Charge Transfer Coefficients and Diffusion Coefficients Determination of Silver, Copper and Rhenium Ions Reductions

Figure S2a, Figure S3a and Figure S4a represent the Tafel plots of the recorded cyclic voltammograms for silver, copper and rhenium ions reduction. As depicted, analysis of slopes enables the determination of the cathodic (α) and the anodic (β) charge transfer coefficients, which are 0.302 and 0.514 for silver ions, 0.727 and 0.460 for copper ions and 0.130 and 0.110 for rhenium ions.

The convoluted currents of cyclic voltammograms for silver, copper and rhenium ions reduction were calculated and presented in Figure S2b, Figure S3a and Figure S4b, respectively.

The diffusion coefficients $D_{Ag^+}$ , $D_{Cu^+}$ and $D_{ReF_8^{2-}}$ , calculated using the relationship (S21), are equal to 5.56 × $10^{-10}$ $m^2$ $s^{-1}$, 2.58 × $10^{-9}$ $m^2$ $s^{-1}$ and 8 ×$10^{-10}$ $m^2$ $s^{-1}$, respectively. In aqueous media, specifically in 1M $HNO_3$, the reported diffusion coefficient for silver ions is 1.46 × $10^{-9}$ $m^2$ $s^{-1}$.[4]

$$m_{max} = nFAD_{M^{n+}}^{1/2} C_{M^{n+}}{}^* \quad (S21)$$

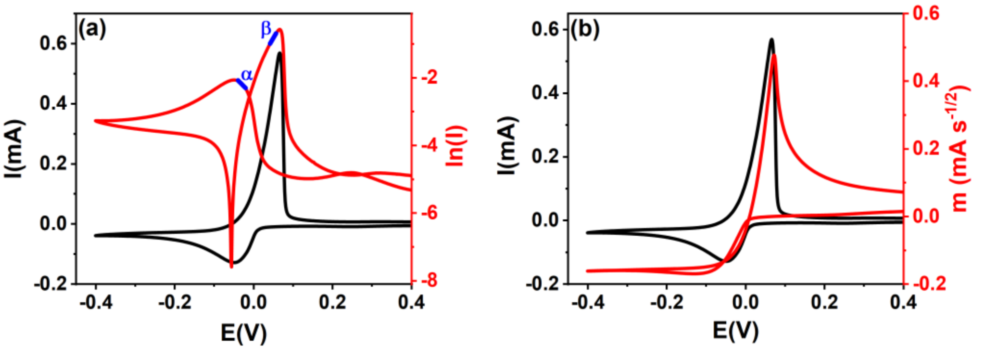


**Figure S2.** Silver ions reduction curves:
(a) Experimental CV (Black) at 50 mV $s^{-1}$ and the corresponding Tafel plots (Red);
(b) Same experimental CV (Black) and its corresponding semi-integral plot (Red)

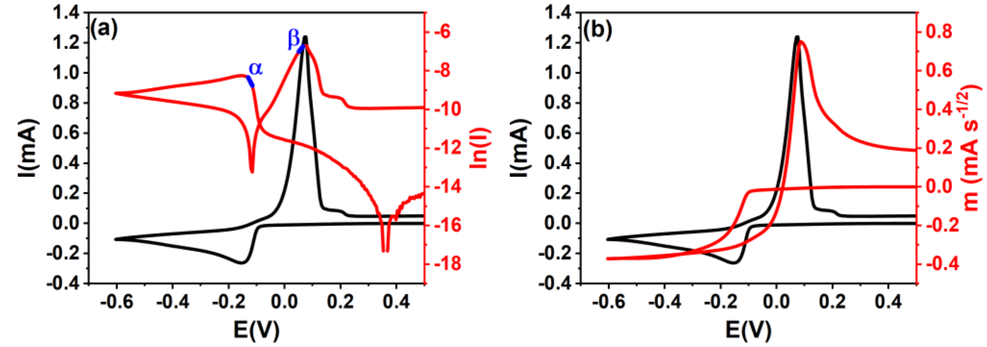


**Figure S3.** Copper ions reduction curves:
(a) Experimental CV (Black) at 100 mV $s^{-1}$ and the corresponding Tafel plots (Red);
(b) Same experimental CV (Black) and its corresponding semi-integral plot (Red)

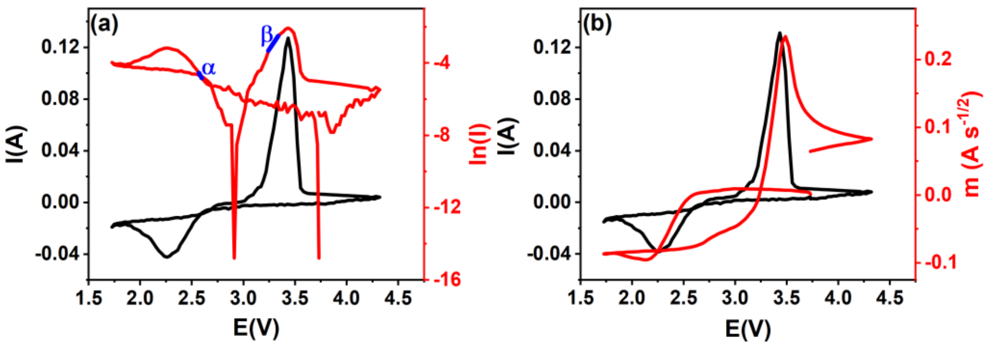


**Figure S4.** Rhenium ions reduction curves:
(a) Experimental cyclic voltammogram (black) recorded at 50 mV $s^{-1}$, adapted from Affoune et al., *J. Appl. Electrochem.*, 2002, **32**, 721–728, https://doi.org/10.1023/A:1016532912889, with permission from Springer Nature, and the corresponding Tafel plot (red);
(b) Same experimental CV (black) and its corresponding semi-integral plot (red).

## IV Theoretical Validation of Interpolation Equations

Table S1 summarizes the theoretical results obtained from Equations (3), (20), and (34). The corresponding conclusions drawn from these calculations are discussed in the main text.

**Table S1.** Theoretical validation of Equations (3), (20), and (34)

| ω | α | β | α+β | Φa | Φc | ΔΦ | ω' | | |
|---|---|---|---|---|---|---|---|---|---|
| | | | | | | | **Eq (3)** | **Eq (20)** | **Eq (34)** |
| **$10^{-6}$** | **0.1** | **0.1** | **0.2** | 128.968 | -128.73 | 257.698 | 1.49E-9 | 0.21E-6 | 1.20E-6 |
| | **0.2** | **0.2** | **0.4** | 67.218 | -66.1 | 133.318 | 1.62E-8 | 1.09E-5 | 1.78E-6 |
| | **0.3** | **0.5** | **0.8** | 28.608 | -44.74 | 73.348 | 0.56E-6 | 1.03E-5 | 1.67E-6 |
| | **0.35** | **0.65** | **1** | 22.378 | -38.57 | 60.948 | 1.59E-6 | 8.10E-6 | 1.59E-6 |
| | **0.60** | **0.60** | **1.2** | 24.028 | -22.95 | 46.978 | 9.85E-6 | 7.09E-6 | 1.19E-6 |
| | **0.75** | **0.75** | **1.5** | 19.498 | -18.51 | 38.008 | 6.12E-4 | 7.49E-6 | 0.74E-6 |
| **$10^{-5}$** | **0.1** | **0.1** | **0.2** | 105.938 | -105.7 | 211.638 | 1.64E-8 | 0.32E-5 | 0.93E-5 |
| | **0.35** | **0.65** | **1** | 18.838 | -31.99 | 50.828 | 0.89E-5 | 2.00E-5 | 0.89E-5 |
| | **0.75** | **0.75** | **1.5** | 16.428 | -15.44 | 31.868 | 2.55E-3 | 1.85E-5 | 0.50E-5 |
| **$10^{-4}$** | **0.1** | **0.1** | **0.2** | 82.908 | -82.67 | 165.578 | 2.63E-7 | 0.43E-4 | 1.04E-4 |
| | **0.35** | **0.65** | **1** | 15.298 | -25.41 | 40.708 | 0.71E-4 | 0.84E-4 | 0.71E-4 |
| | **0.75** | **0.75** | **1.5** | 13.358 | -12.37 | 25.728 | 9.08E-3 | 0.97E-4 | 0.49E-4 |
| **$10^{-3}$** | **0.1** | **0.1** | **0.2** | 59.868 | -59.64 | 119.508 | 8.08E-6 | 0.82E-3 | 1.46E-3 |
| | **0.2** | **0.2** | **0.4** | 32.678 | -31.56 | 64.238 | 2.26E-5 | 1.29E-3 | 1.35E-3 |
| | **0.3** | **0.5** | **0.8** | 14.788 | -21.72 | 36.508 | 0.37E-3 | 0.72E-3 | 0.98E-3 |
| | **0.35** | **0.65** | **1** | 11.748 | -18.83 | 30.578 | 0.86E-3 | 0.77E-3 | 0.86E-3 |
| | **0.60** | **0.60** | **1.2** | 12.518 | -11.44 | 23.958 | 3.71E-3 | 0.47E-3 | 0.73E-3 |
| | **0.75** | **0.75** | **1.5** | 10.288 | -9.3 | 19.588 | 2.65E-2 | 1.93E-3 | 0.73E-3 |
| **$10^{-1}$** | **0.1** | **0.1** | **0.2** | 15.428 | -14.41 | 29.838 | 0.27E-1 | 0.81E-1 | 1.14E-1 |
| | **0.2** | **0.2** | **0.4** | 10.088 | -8.58 | 18.668 | 0.38E-1 | 0.96E-1 | 1.32E-1 |
| | **0.3** | **0.5** | **0.8** | 5.648 | -6.06 | 11.708 | 0.80E-1 | 0.97E-1 | 1.11E-1 |
| | **0.35** | **0.65** | **1** | 4.708 | -5.4 | 10.108 | 1.00E-1 | 0.98E-1 | 0.48E-1 |
| | **0.60** | **0.60** | **1.2** | 4.888 | -3.71 | 8.598 | 1.35E-1 | 0.90E-1 | 0.85E-1 |
| | **0.75** | **0.75** | **1.5** | 4.188 | -3.11 | 7.298 | 2.20E-1 | 1.01E-1 | 0.69E-1 |
| **$2\times10^{-1}$** | **0.1** | **0.1** | **0.2** | 11.288 | -10.25 | 21.538 | 0.53E-1 | 1.16E-1 | 2.16E-1 |
| | **0.2** | **0.2** | **0.4** | 7.578 | -6.06 | 13.638 | 0.78E-1 | 1.39E-1 | 2.48E-1 |
| | **0.3** | **0.5** | **0.8** | 4.468 | -3.97 | 8.438 | 1.54E-1 | 1.63E-1 | 2.12E-1 |
| | **0.35** | **0.65** | **1** | 3.778 | -3.48 | 7.258 | 1.90E-1 | 1.74E-1 | 1.92E-1 |
| | **0.60** | **0.60** | **1.2** | 3.868 | -2.66 | 6.528 | 2.24E-1 | 1.55E-1 | 1.51E-1 |
| | **0.75** | **0.75** | **1.5** | 3.348 | -2.25 | 5.598 | 3.30E-1 | 1.65E-1 | 1.21E-1 |
| **$10^{0}$** | **0.1** | **0.1** | **0.2** | 6.548 | -5.76 | 12.308 | 0.13E0 | 0.17E0 | 0.74E0 |
| | **0.2** | **0.2** | **0.4** | 4.358 | -3.16 | 7.518 | 0.22E0 | 0.21E0 | 0.82E0 |
| | **0.3** | **0.5** | **0.8** | 2.738 | -1.79 | 4.528 | 0.42E0 | 0.29E0 | 0.61E0 |
| | **0.35** | **0.65** | **1** | 2.348 | -1.5 | 3.848 | 0.52E0 | 0.34E0 | 0.53E0 |
| | **0.60** | **0.60** | **1.2** | 2.248 | -1.27 | 3.518 | 0.58E0 | 0.35E0 | 0.45E0 |
| | **0.75** | **0.75** | **1.5** | 1.948 | -1.06 | 3.008 | 0.78E0 | 0.40E0 | 0.39E0 |
| **$10^{1}$** | **0.1** | **0.1** | **0.2** | 5.128 | -4.43 | 9.558 | 0.19 | 0.19 | 1.62 |
| | **0.2** | **0.2** | **0.4** | 3.288 | -2.25 | 5.538 | 0.35 | 0.24 | 1.72 |
| | **0.3** | **0.5** | **0.8** | 2.048 | -1.15 | 3.198 | 0.68 | 0.35 | 1.06 |
| | **0.35** | **0.65** | **1** | 1.748 | -0.93 | 2.678 | 0.85 | 0.42 | 0.89 |
| | **0.60** | **0.60** | **1.2** | 1.558 | -0.78 | 2.338 | 1.01 | 0.48 | 0.88 |
| | **0.75** | **0.75** | **1.5** | 1.328 | -0.63 | 1.958 | 1.34 | 0.57 | 0.92 |

## V MATLAB Code for log(ω) Interpolation Equations Calculation

The Matlab code corresponding to Equations (3), (20) and (34):

```
dep_value = ;  % replace with your desired  dep dimensionless value
alpha_value = ;  % replace with your desired alpha value
beta_value = ;   %where alpha+beta=1
beta_0_value =1-alpha_value;  %where alpha+beta=1
n_value= ;
F_value=96485.31;
R_value=8.314;
T_value= ;
v_value= ;
t2_value= ; %where alpha+beta=1(experimental)of the anodic peak: from the I=0 to the
the anodic peak

alpha=alpha_value; beta=beta_value; dep=dep_value;

logw1_result = customSolveEquation2(dep_value, alpha_value, beta_value);
logw2_result = customSolveEquation3(dep_value, alpha_value, beta_value,...
               beta_0_value,n_value,F_value,R_value,T_value,v_value,t2_value);

function logw1 = customSolveEquation2(dep, alpha, beta)

    % Define the equation
    equation = @(logw1) 1 ./ ((0.0012 + 0.352 *exp((-logw1)/(-0.4862))) + ...
                     ((0.3045* exp(0.2641*logw1)) * alpha) + ...
                     ((-0.3136*exp(0.2741*logw1)) * alpha^2)) - dep +...
                     (( 0.27594+1.43996*logw1-0.01107* logw1 ^2)+...
                     ( -5.28228-9.8649*logw1+0.04393* logw1 ^2)*alpha+...
                     ( 7.30133+19.49543*logw1-0.31554* logw1 ^2)* alpha^2)+...
                     (( -58.60695-88.52844*logw1-12.87437* logw1 ^2)+...
                     ( 155.89195+214.46174*logw1+53.23083* logw1 ^2)* alpha +...
                     ( -128.3385-175.8604*logw1-43.75774* logw1 ^2)* alpha^2)*...
                     exp((( -0.70606+4.20589*logw1+0.80246* logw1 ^2)+...
                     ( -5.06505-5.5916*logw1-1.31285* logw1 ^2)* alpha +...
                     ( 4.59224+4.88175*logw1+1.12871* logw1 ^2)* alpha^2)*beta);


    % Set options for lsqnonlin
    options = optimoptions('lsqnonlin', 'Display','off', 'FunctionTolerance', 1e-12,...
              'OptimalityTolerance', 1e-12, 'MaxIterations', 1000);

    % Set lower and upper bounds
    lb = -6;
    ub = 6;

    % Generate a random initial guess within the specified range
    initial_guess = (ub - lb) * rand() + lb;

    % Use lsqnonlin for constrained optimization
    logw1_solution = lsqnonlin(equation, initial_guess, lb, ub, options);

    if ~isempty(logw1_solution)
        logw1 = logw1_solution;
    else
        error('No solution found.');

    end

end

function logw2 = customSolveEquation3(dep, alpha, beta, beta_0,n,F,R,T,v,t2)
```

```
    % Define the equation
    equation = @(logw2) 1 ./ ((0.0012 + 0.352 *exp((-logw2)/(-0.4862))) + ...
                     ((0.3045* exp(0.2641*logw2)) * alpha) + ...
                     ((-0.3136*exp(0.2741*logw2)) * alpha^2)) - dep +...
                     ((v*(t2-((R*T)/(beta_0*n*F*v))*...
                     log(((beta_0*(exp((beta*n*F*v*t2)/(R*T)))-
1)/beta)+1))*(n*F/(R*T))));


    % Set options for lsqnonlin
    options = optimoptions('lsqnonlin', 'Display','off', 'FunctionTolerance', 1e-12,...
                           'OptimalityTolerance', 1e-12, 'MaxIterations', 1000);

    % Set lower and upper bounds
    lb = -6;
    ub = 6;

    % Generate a random initial guess within the specified range
    initial_guess = (ub - lb) * rand() + lb;

    % Use lsqnonlin for constrained optimization
    logw2_solution = lsqnonlin(equation, initial_guess, lb, ub, options);

    if ~isempty(logw2_solution)
        logw2 = logw2_solution;
    else
        error('No solution found.');
    end
end
```

**Nomenclature**

| | |
|---|---|
| A | : Surface area of electrode / $m^2$ |
| $C_{M^{n+}}$ | : Concentration of the metallic ions / mmol $L^{-1}$ |
| $C_M$ | : Concentration of the metal species / mmol $L^{-1}$ |
| $C_{M^{n+}}$ (0,t) | : Concentration of the metallic ions at the electrode surface / mmol $L^{-1}$ |
| $C_M$ (0,t) | : Concentration of the metal species at the electrode surface / mmol $L^{-1}$ |
| $C^*_{M^{n+}}$ | : Concentration of the metallic ions at t = 0 / mmol $L^{-1}$ |
| $C^*_M$ | : Concentration of the metal species at t = 0 / mmol $L^{-1}$ |
| $C^0$ | : Standard concentration (1 mol $L^{-1}$) |
| D | : Diffusion coefficient / $m^2$ $s^{-1}$ |
| E | : Electrode potential / V |
| $E^0$ | : Standard potential / V |
| $E_{eq}$ | : Equilibrium potential / V |
| $E_a$ | : Anodic peak potential / V |
| $E_c$ | : Cathodic peak potential / V |
| $E_\lambda$ | : Switching potential / V |
| $\Delta E_p$ | : Peak-to-peak potential separation / V |
| F | : Faraday's constant / C $mol^{-1}$ |
| $J_{M^{n+}}(0, t)$ | : Flux of metallic ions / mol.$m^{-2}$.$s^{-1}$ |
| I | : Electrode current / A |
| $I_{pa}$ | : Anodic peak current / A |
| $I_{pc}$ | : Cathodic peak current / A |
| $k^0$ | : Standard heterogeneous rate constant / m $s^{-1}$ |
| n | : Number of electrons / unitless |
| R | : Universal gas constant / j $mol^{-1}$ $K^{-1}$ |
| T | : Absolute temperature / K |
| t | : Time / s |
| $v$ | : Scan rate / v $s^{-1}$ |
| $\omega$ | : Dimensionless rate constant / unitless |
| $\alpha$ | : Cathodic charge transfer coefficient / unitless |
| $\beta$ | : Anodic charge transfer coefficient / unitless |
| $\chi$ | : Dimensionless current / unitless |
| $\Phi$ | : Dimensionless potential / unitless |
| $\Delta\Phi$ | : Dimensionless peak-to-peak potential separation / unitless |
| $\Delta\Phi a$ | : Dimensionless anodic peak potential / unitless |
| $\Delta\Phi c$ | : Dimensionless cathodic peak potential / unitless |
| $\eta_p$ | : Dimensionless peak potential / unitless |
| $\Delta\eta_p$ | : Dimensionless anodic peak potentials difference / unitless |
| $\sigma$ | : Dimensionless scan rate / unitless |

## References

1 I. Atek, S. Maye, H. H. Girault, A. M. Affoune and P. Peljo, *Journal of Electroanalytical Chemistry*, 2018, **818**, 35–43.
2 Y. Liu, H. Ren, T. Yin, D. Yang, Z. Chai and W. Shi, *Electrochimica Acta*, 2019, **326**, 134971.
3 R. S. Nicholson, *Anal. Chem.*, 1965, **37**, 1351–1355.
4 D. Liu, D. Krulic, H. Groult and N. Fatouros, *Journal of Electroanalytical Chemistry*, 2016, **775**, 91–104.
